# Synergistic effects of dimethyloxallyl glycine and recombinant human bone morphogenetic protein-2 on repair of critical-sized bone defects in rats

**DOI:** 10.1038/srep42820

**Published:** 2017-02-23

**Authors:** Xin Qi, Yang Liu, Zhen-yu Ding, Jia-qing Cao, Jing-huan Huang, Jie-yuan Zhang, Wei-tao Jia, Jing Wang, Chang-sheng Liu, Xiao-lin Li

**Affiliations:** 1 Department of Orthopaedic Surgery, Shanghai Jiao Tong University Affiliated Sixth People’s Hospital, Shanghai 200233, PR China; 2 Engineering Research Center for Biomedical Materials of Ministry of Education, East China University of Science and Technology, Shanghai 200237, PR China; 3 The State Key Laboratory for Bioreactor Engineering, East China University of Science and Technology, Shanghai 200237, PR China; 4 Key Laboratory for Ultrafine Materials of Ministry of Education, East China University of Science and Technology, Shanghai 200237, PR China.

## Abstract

In bone remodeling, osteogenesis is closely coupled to angiogenesis. Bone tissue engineering using multifunctional bioactive materials is a promising technique which has the ability to simultaneously stimulate osteogenesis and angiogenesis for repair of bone defects. We developed mesoporous bioactive glass (MBG)-doped poly(3-hydroxybutyrate-co-3-hydroxyhexanoate) (PHBHHx) composite scaffolds as delivery vehicle. Two bioactive molecules, dimethyloxalylglycine (DMOG), a small-molecule angiogenic drug, and recombinant human bone morphogenetic protein-2 (rhBMP-2), an osteoinductive growth factor, were co-incorporated into the scaffold. The synergistic effects of DMOG and rhBMP-2 released in the composite scaffolds on osteogenic and angiogenic differentiation of hBMSCs were investigated using real-time quantitative polymerase chain reaction and western blotting. Moreover, *in vivo* studies were conducted to observe bone regeneration and vascular formation of critical-sized bone defects in rats using micro-computed tomography, histological analyses, Microfil^®^ perfusion, fluorescence labeling, and immunohistochemical analysis. The results showed that DMOG and rhBMP-2 released in the MBG-PHBHHx scaffolds did exert synergistic effects on the osteogenic and angiogenic differentiation of hBMSCs. Moreover, DMOG and rhBMP-2 produced significant increases in newly-formed bone and neovascularization of calvarial bone defects in rats. It is concluded that the co-delivery strategy of both rhBMP-2 and DMOG can significantly improve the critical-sized bone regeneration.

In the clinic, bone defects caused by trauma, severe infection, tumor resection, and skeletal abnormalities are major challenges in orthopedic surgery^1^. About 2.2 million bone grafts are used annually worldwide^2^. Therapies for enhancing osteogenesis remain a critical challenge in reconstruction of large bone defects due to insufficient osteogenic capacity of biomaterials. Although most available strategies are currently used to treat defects, such as autologous bone transplantation, allogeneic bone transplantation, or combinations of biomaterials and growth factors or cells, autologous bone grafting remains the gold standard for the clinical treatment^3^,^4^. However, complications such as disease transmission, donor site morbidity, and high costs remain unresolved. The introduction of osteogenic factors is an efficient approach for improving bone regeneration^5^. A variety of osteogenic factors, especially bone morphogenetic protein-2 (BMP-2), the most notable osteogenic cytokine^6^,^7^,^8^, are involved in promoting osteogenesis in the process of new bone formation. Recombinant human BMP-2 (rhBMP-2) products have been approved by the US Food and Drug Administration (FDA) and the European Medicines Agency and have had some clinical success for bone repair^9^,^10^,^11^. However, clinical applications of rhBMP-2 products are limited by factors such as the short half-life of the protein and its easy deactivation. Therefore, design of a releasing system, as well as prolongation of the half-life, are needed before a biomaterial scaffold can be developed for the treatment of bone defects.

In the processes of bone development and regeneration, osteogenesis is closely related to angiogenesis. The vasculature provides a source of nutrients, oxygen, and metabolic substrates, as well as access for circulating cells that help to support tissue regeneration. Blood vessel invasion is a critical event in the replacement of calcified cartilage by bone and in the formation of bone marrow^2^,^12^,^13^. Xiao *et al*.^14^ showed that bone marrow stromal cells that expressed both BMP-2 and vascular endothelial growth factor (VEGF)-165 enhanced bone regeneration. Qu *et al*.^15^ also demonstrated that angiogenesis and osteogenesis enhanced by *ex vivo* gene therapy using basic fibroblast growth factor could serve as a form of bone tissue engineering for the reconstruction of calvarial defects. However, these approaches still have many limitations, such as high cost and easy deactivation, and ways to promote the angiogenesis and vascularization of these bone constructs remains a big challenge.

Recently, much attention has been paid to dimethyloxalylglycine (DMOG), a small-molecule drug known to be a cell-permeable, competitive inhibitor of hypoxia-inducible factor prolyl hydroxylase (HIF-PH)^5^,^16^. At normal oxygen tensions, HIF-PH hydroxylate is a specific proline residue in HIF-1a which can bind HIF to the von Hippel–Lindau tumor suppressor, leading to degradation of HIF^17^. HIF-1a is a key transcriptional regulator of vasculogenesis and angiogenesis^18^,^19^. DMOG can inhibit the effect of HIF-PH, and thus stabilize the expression of HIF-1a in cells. Therefore, use of DMOG, which acts as a pro-angiogenic compound, is expected to be an alternative strategy for enhancing angiogenesis. As a result, DMOG has been successfully used to induce angiogenesis in ischemic skeletal muscles^20^ and to enhance bone healing^21^ by improving angiogenesis. Wu *et al*.^17^ showed that delivery of DMOG in mesoporous bioactive glass (MBG) scaffolds could improve the angiogenesis and osteogenesis of human bone marrow stromal cells (hBMSCs). Min *et al*.^22^ have reported that 3D-printed DMOG-loaded mesoporous bioactive glass (MBG)/poly(3-hydroxybutyrate-co-3-hydroxyhexanoate) (PHBHHx) composite scaffolds can promote blood vessel growth and bone regeneration *in vivo*.

Motivated by these previous investigations, we propose the concept of combination treatments of osteogenesis and angiogenesis via simultaneous co-delivery of rhBMP-2 and DMOG. A composite PHBHHx-MBG scaffold, composed of biodegradable PHBHHx and MBG, was selected as the vehicle for both osteogenic growth factor and angiogenic drug molecules, owing to the advantages of its good biocompatibility, as well as the favorable loading and releasing behavior of its mesoporous structure.

On the basis of the above rationale, PHBHHx-MBG (PHMG) complex scaffolds incorporating a dual delivery system of rhBMP-2 and DMOG were fabricated. The microstructure of the complex scaffold, as well as the cell affinity and *in vitro* releasing profiles of DMOG and BMP-2, were well characterized. The effects of dual delivery on osteogenic- and angiogenic-related gene expression were investigated. After the scaffolds were implanted into a critical-sized calvarial defect model of a rat, bone regeneration and blood formation were qualitatively and quantitatively evaluated using micro-computed tomography (micro-CT), sequential fluorescent labeling, and histological analysis. Although the individual roles of BMP-2 and DMOG are clear, reciprocal effects occurred in this dual delivery system, which led to significant augmentation of new bone and microvessel formation in the defects (Scheme 1). Therefore, the aim of this study was to depict the synergistic interactions between sustained release of DMOG and rhBMP-2, in order to reveal whether the combination protocol could expedite bone regeneration in critical-sized defects. This study has the following four groups: pure MBG-PHBHHx scaffolds were named PHMG, BMP-2 + MBG-PHBHHx scaffolds were named PHMB, DMOG + MBG-PHBHHx scaffolds were named PHMD and BMP-2 + DMOG + MBG-PHBHHx scaffolds were named PHMBD.

## Results

### Characterization of hierarchical PHMG composite scaffolds

**Figure 1:**
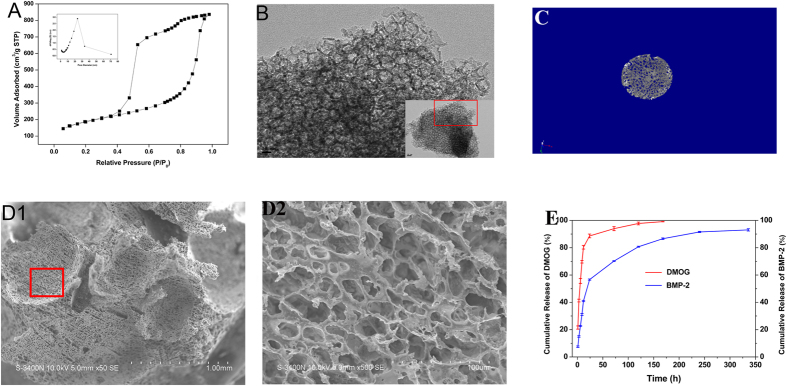
(**A**) Nitrogen adsorption–desorption isotherm analysis of MBG. The inset shows a plot of the pore size distribution of MBG. (**B**) TEM analysis of the prepared MBG. The inset shows a higher-magnification image. (**C**) Micro-CT analysis of the prepared MBG-PHBHHx composite scaffold. (**D**) SEM analysis of the prepared MBG-PHBHHx composite scaffold. D1: SEM image at a magnification of ×50. D2: SEM image of the red box shown in D1 at a magnification of ×500. (**E**) Release of DMOG and rhBMP-2 in MBG-PHBHHx composite scaffolds.

Composite PHMG scaffolds with hierarchical pore structures were fabricated for their improved cellular affinity compared to a pure polymeric matrix, as well as their toughness compared to the fragile porous MBG. A mesoporous microstructure was afforded by the MBG powders, which were synthesized using non-ionic block copolymers as structure-directing agents via an EISA process as previously reported. A TEM image (Fig. 1B) reveals the disordered mesoporous structure, with a comparatively large specific surface area of approximately 583.928 m^2^/g as determined by the BET method. Other parameters, such as pore volume and pore size, can be calculated from the nitrogen sorption isotherms to be approximately 1.243 cm^3^/g and 23.722 nm, respectively (Fig. 1A). Interconnected porous architectures could be observed in SEM images. The reconstituted micro-CT scan shown in Fig. 1C confirms the high interconnectivity, with a porosity of approximately 80%. As indicated in Fig. 1D, the macroporous structure produced by the porogen has open pore channels of approximately 450–900 μm. Notably, an abundant smaller-sized pores with morphology of 20–50 μm distributed homogenously on the frame walls of the strut can be observed at a higher magnification, which is derived from the phase separation of 1,4-dioxane. As to the mechanical strength, PHMG scaffold possessed suitable compressive strength of about 9.91 ± 0.19 × 10^−3^ MPa.

### Release behaviors of DMOG and rhBMP-2 in porous PHMG composite scaffolds

The release behaviors of both DMOG and rhBMP-2 are displayed in Fig. 1E. As a small-molecule drug, DMOG is apt to diffuse from the skeleton of the scaffold and liberate rapidly. Although burst release was displayed, DMOG was released for nearly 7 days until it had been almost completely released. In comparison, release of rhBMP-2 occurred in a more slowly released mode. As exhibited in Fig. 1E, retarded release of approximately 40% of rhBMP-2 was observed for the first 12 h and, in the later stage, sustained release continued until 14 days. The rough and porous topological structure facilitates the adsorption of rhBMP-2. Particularly, the dispersed approximately 20–50 μm smaller-sized pores play an important role in prolonging release of BMP-2.

### Cell attachment and viability

**Figure 2:**
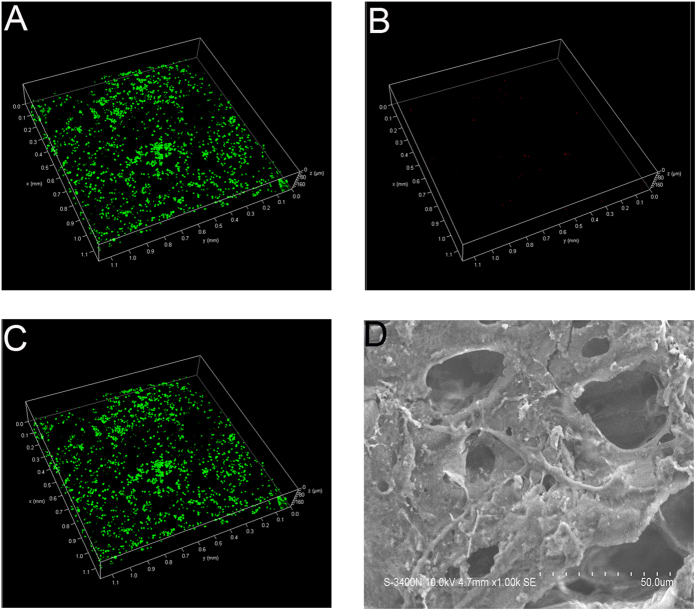
Live/dead staining of hBMSCs cultured for 24 h. (**A**) Live cells (green). (**B**) Dead cells (red). (**C**) Superimposition of A and B. (**D**) The morphology of RAW 264.7 cells on scaffolds.

Visualized live/dead fluorescent staining was employed to estimate the cell affinity of the composite scaffold. Live hBMSCs were stained green and appeared to have adhered, while dead cells with damaged membranes were stained red. As expected, the composite scaffold was shown to support growth and adhesion of cells. As shown in Fig. 2A–C, dead cells (red spots) could hardly be observed on the scaffolds, indicating the good attachment of the matrix. The results of SEM showed the RAW 264.7 cells presented well-spread morphology on scaffolds (Fig. 2D).

### Effects of DMOG and rhBMP-2 co-delivery on osteogenic and angiogenic gene expression of hBMSCs

Real-time qRT-PCR analysis was conducted to examine the osteogenic and angiogenic expression of PHMB and PHMBD using hBMSCs. In general, the relative expression of bone-related genes was elevated with the increase in PHMB and PHMBD groups. In addition, the VEGF expression level of hBMSCs cultured with PHMD was significantly higher than those cultured with PHMG.

**Figure 3:**
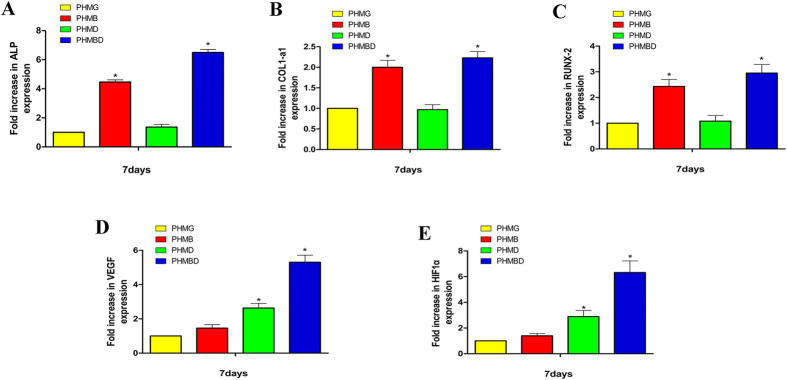
mRNA expression levels of (**A**) ALP, (**B**) COL1-a1, (**C**) RUNX-2, (**D**) VEGF, and (**E**) HIF-1α as measured by qRT-PCR at 7 days. (*P < 0.05).

RT-qPCR analysis revealed that DMOG released in the PHMD group barely increased the bone-related mRNA expression levels of ALP (Fig. 3A), COL1 (Fig. 3B), and RUNX-2 (Fig. 3C). mRNA expression was apparently higher in the PHMB and PHMBD groups compared with the other two groups. The mRNA expression of ALP in the PHMBD group was more than 6 times that in the PHMG group (*P* < 0.05). There was a significant difference in mRNA expression in the PHMBD group compared with the PHMD and PHMB groups (*P* < 0.05), and there was also a significant difference between the PHMB and PHMD groups (*P* < 0.05). Similar tendency was occurred in the mRNA expression of RUNX-2, PHMBD group appeared to be the highest. There was a significant difference in mRNA expression of RUNX-2 in the PHMBD group compared with the PHMD and PHMB groups (*P* < 0.05), and there was also a significant difference between the PHMB and PHMD groups (*P* < 0.05). mRNA expression of COL1 in PHMBD group differed significantly compared with the other groups (*P* < 0.05), and there was also a significant difference between the PHMB and PHMD groups (*P* < 0.05). The above results suggested that delivery of DMOG and rhBMP-2 in the PHMBD group synergistically increased osteogenic expression of hBMSCs, and this effect was statistically significant (*P* < 0.05).

The mRNA expression of VEGF (Fig. 3D) and HIF-1a (Fig. 3E) showed that DMOG in the PHMD and PHMBD groups significantly increased the expression levels of HIF-1a and VEGF. mRNA expression of VEGF in the PHMBD group appeared to be higher compared with that in the PHMG group (*P* < 0.05), there was a significant difference in mRNA expression of VEGF in the PHMBD group compared with the PHMD and PHMB groups (*P* < 0.05), and there was also a significant difference between the PHMD and PHMB groups (*P* < 0.05). As expected, HIF-1a expression under normoxic conditions was apparently upregulated in the PHMD and PHMBD groups, and there was a significant difference in the PHMBD group compared with the PHMD group (*P* < 0.05). It is noticeable that synergistic interactions may exist between DMOG and BMP-2. Neither DMOG nor BMP-2 has reciprocal effects on osteogenesis and angiogenesis. Nevertheless, significant enhancements could be observed in the co-delivery group. This indicates that combination treatment with BMP-2 and DMOG may enhance both osteogenesis and angiogenesis.

**Figure 4:**
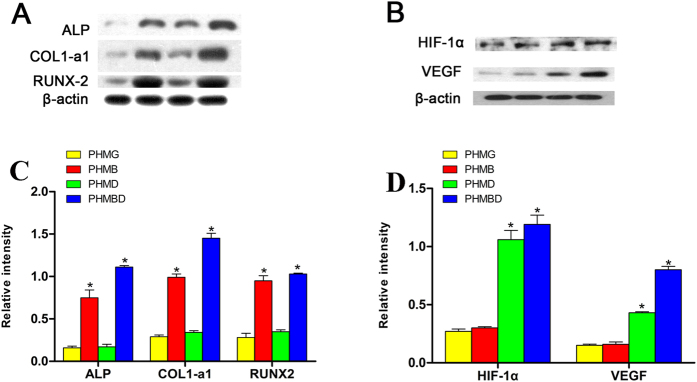
Protein levels of (**A**) ALP, RUNX-2, and COL1-a1 and (**B**) HIF-1α and VEGF as determined by western blotting, (**C**) the relative intensity of A, (**D**) the relative intensity of (**B**), *(*P* < 0.05).

Western blotting analysis of osteogenic and angiogenic expression of hBMSCs was further investigated (Fig. 4). The results revealed that bone-related protein expression of hBMSCs was increased in the PHMB and PHMBD groups, but not up-regulated in the PHMG and PHMD groups. As previously reported, DMOG can significantly increase the expression levels of pro-angiogenic factors such as HIF-1a and VEGF, while BMP-2 can enhance the expression of osteoblast markers. Interestingly, combined delivery of BMP-2 and DMOG apparently increased the expression of both pro-angiogenic and osteogenic molecules, which is consistent with the RT-qPCR analysis. These results confirm that both DMOG and BMP-2 can exert their angiogenic and osteogenic potency, respectively, in the presence of each other, implying that a synergistic effect exists when the two substances are used in combination.

### Micro-CT analysis of bone regeneration

**Figure 5:**
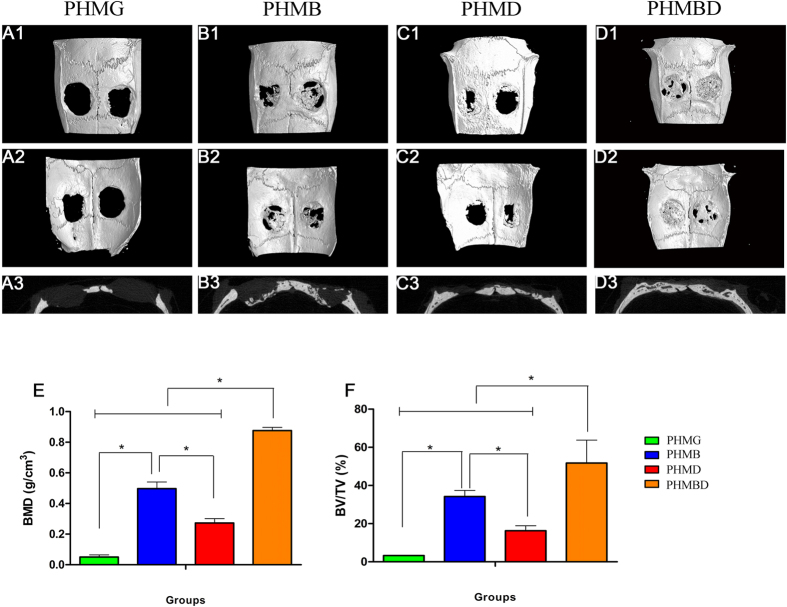
Representative 3D reconstructions of superficial (A1–D1), interior (A2–D2), and sagittal images (A3–D3) of calvarial bone defects taken at 8 weeks after implantation. Morphometric analysis of (**E**) BMD and (**F**) BV/TV as determined by micro-CT for each group at 8 weeks. ^*^P < 0.05.

The micro-CT images of the 3D morphology and 2D slices of the repaired calvarial bones at week 8 are presented in Fig. 5. Figure 5A1–D2 depict the 3D morphological images of the newly-formed calvarial bones obtained using micro-CT reconstruction. In the sagittal view (Fig. 5A3–D3), little bone growth was observed in the defect in the PHMG group. Attributable to the improved proangiogenic effect, the PHMD group showed increased new bone formation. As for the PHMB group, with the help of osteoinductive growth factor, newly-formed bone was apparently augmented. However, small gaps at the fringes of the scaffold and in the center with less bone formation still existed. The combined treatment used in the PHMBD group meant that the defect was almost completely filled with new calvarium, and the interfaces between the scaffold and bone tissues were connected. The local BMDs were markedly the highest at 0.876 ± 0.021 g/cm^3^ (Fig. 5E), and there was a significant difference between the PHMB and PHMD groups (*P* < 0.05). Moreover, BV/TV showed the same tendency as the BMD levels (Fig. 5F), i.e. there was a significant difference in the PHMBD group compared with the PHMD and PHMB groups (*P* < 0.05), and also a significant difference between the PHMB and PHMD groups (*P* < 0.05). These results indicate that DMOG and rhBMP-2 released in the PHMBD group can synergistically improve bone regeneration compared with the other groups, which was consistent with the results of the real-time qRT-PCR and western blotting analysis.

### Fluorochrome labeling histomorphometric analysis

**Figure 6:**
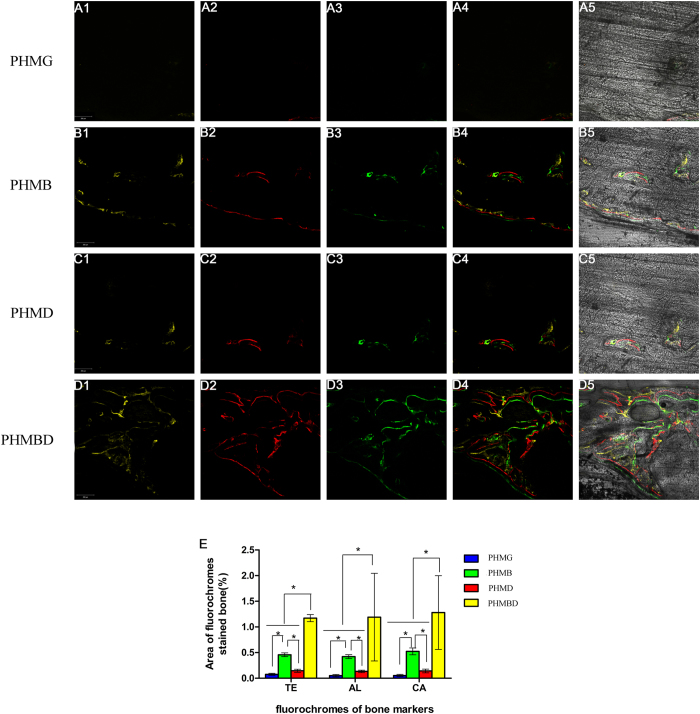
Images A1–D1 in yellow represent TE, images A2–D2 in red represent AL, and images A3–D3 in green represent CA indicating bone formation and mineralization at 2, 4, and 6 weeks after operation, respectively. (A4–D4): Merged images of the three fluorochromes. (A5–D5): Merged images of the three fluorochromes together with a bright-field CLSM image. (**E**) The percentages of TE, AL, and CA staining for each group assessed at week 8 after implantation by histomorphometric analysis. *P < 0.05. Scale bar: 200 μm.

The fluorescent labeling analysis was performed at consecutive 2-week intervals of 2, 4, and 6 weeks, so as to observe the formation of new bone over different periods. As revealed in Fig. 6, at 2 weeks the percentage of TE labeling (yellow) in the PHMBD group (1.172 ± 0.068%) was larger than that in the PHMG (0.076 ± 0.021%), PHMD (0.146 ± 0.03%), and PHMB groups (0.458 ± 0.036%) (*P* < 0.05). At 4 weeks, the highest percentage of AL labeling (red) was observed in the PHMBD group (1.19 ± 0.854%), but there was also a significant difference between the PHMB (0.422 ± 0.038%), PHMD (0.134 ± 0.024%), and PHMG groups (0.05 ± 0.023%) (*P* < 0.05). At 6 weeks, the percentage of CA labeling (green) in the PHMBD group (1.280 ± 0.718%) was significantly higher than that in the PHMG (0.054 ± 0.021%), PHMD (0.142 ± 0.036%), and PHMB groups (0.524 ± 0.067%) (*P* < 0.05), but there was also a significant difference between the PHMB group and the other two groups (*P* < 0.05). In particular, all three fluorescent stains appeared throughout the defect at 6 weeks in the PHMD group, demonstrating the rapid bone regeneration achieved using the combination protocol.

### Neovascularization of calvarial defects

**Figure 7:**
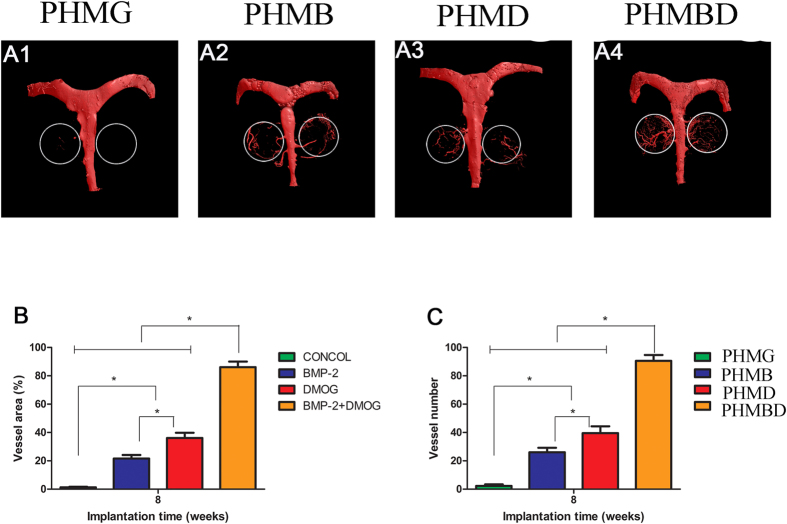
(A1–A4) New blood vessel formation in the calvarial defects in the form of 3D reconstructed images. Morphometric analysis was performed to evaluate (**B**) local blood vessel area and (**C**) blood vessel number in the bone defects. *P < 0.05.

Neovascularization in the defect area was detected by Microfil^®^ perfusion at 8 weeks, and subsequently imaged with micro-CT scanning, as displayed in Fig. 7. Three-dimensional micro-CT images showed that newly-generated microvessels in the defect were evident in the PHMBD and PHMD groups, whereas blood vessels could rarely be seen in the blank scaffold group. Although a few vessels were observed in the PHMB group, the capillaries were discrete. It was noticeable that in the significantly dense and interconnected capillary network generated in the PHMBD group, the microvessels grew along the round edge of the scaffolds. Quantification of the new blood vessel area (Fig. 7B) and vessel numbers (Fig. 7C) verified the above results. There was a significant difference in newly-formed blood vessel areas between the PHMBD (86.09 ± 3.989%), PHMD (36.11 ± 3.687%), PHMB (21.648 ± 2.459%), and PHMG groups (1.265 ± 0.415%) (*P* < 0.05), and the PHMBD group showed the largest area of neovascularization. These results corresponded to DMOG release.

### Histological analysis

**Figure 8:**
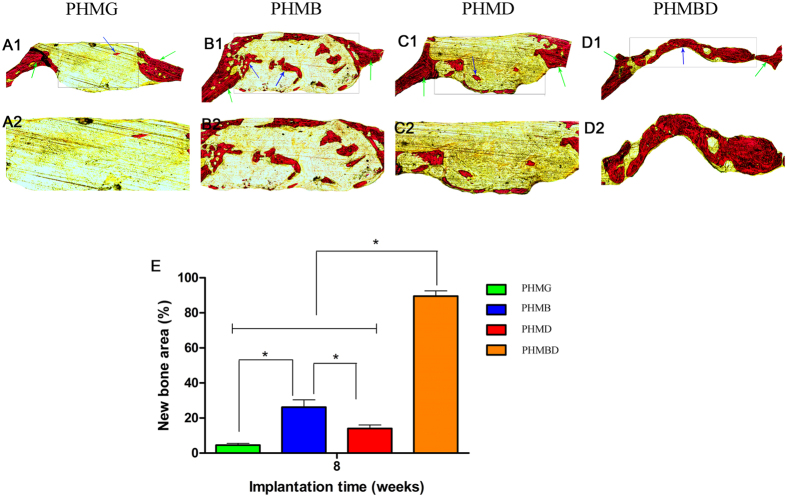
The un-decalcified craniums were sectioned and stained with van Gieson’s picrofuchsin (A1–D1). New bone area is shown in red. (**E**) The percentage of new bone area was assessed at 8 weeks after implantation by histomorphometric analysis. *P < 0.05.

Analysis of Van Gieson’s picrofuchsin staining clearly showed that barely any new bone formation was found in the PHMG group(Fig. 8A). Only a small amount of new bone formation was observed in the PHMD group (Fig. 8C). In the PHMB group (Fig. 8B), the ingrowth of new bone formation was evident in the central area of the defects as well as in the peripheral area near the pre-existing bones. In the PHMBD group, bone formation was most active; the newly-formed bone tissues were relatively thick and almost covered the area of the defect (Fig. 8D). The histomorphometric results showed that the percentage of new bone area was significantly greater in the PHMBD group (89.5 ± 3.017%) compared with the PHMG (4.50 ± 1.049%), PHMB (26.167 ± 4.167%), and PHMD groups (14.00 ± 2.00%) (*P* < 0.05) (Fig. 8E).

### IHC analysis

**Figure 9:**
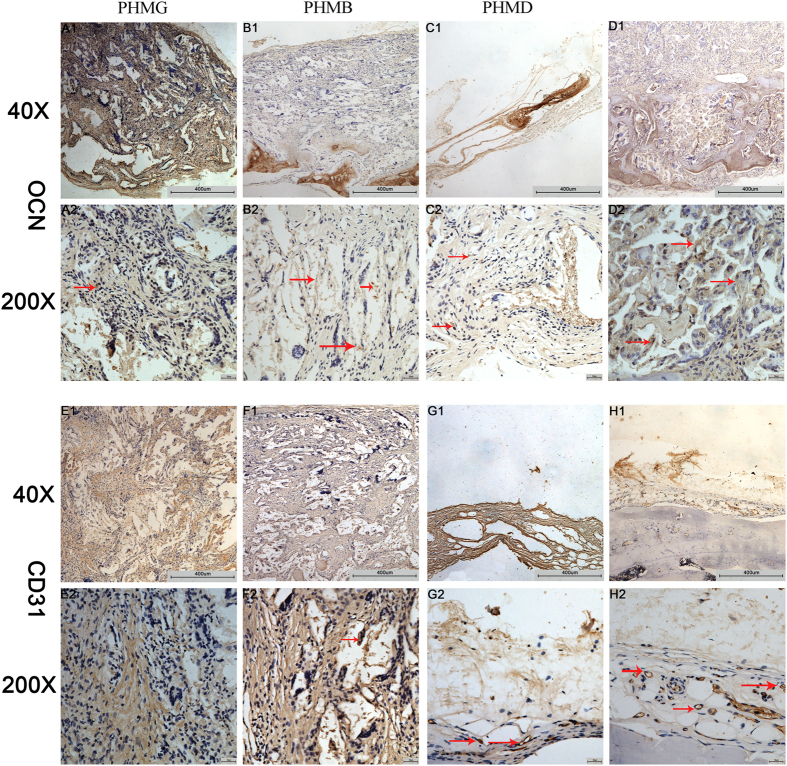
IHC analysis of (**A**–**D**) OCN and (**E**–**H**) CD31 was used to detect osteogenesis and angiogenesis in specimens. The brown color represents positive staining of OCN and CD31. Scale bars: 200 μm and 50 μm.

The osteogenic marker OCN was detected by IHC staining of decalcified craniums (Fig. 9A–D). The results showed that there was almost no obvious positive staining for OCN (Fig. 9A1,A2) in the PHMG group. Positive brown staining for OCN was more apparent in the PHMB (Fig. 9B1,B2) and PHMBD groups (Fig. 9D1,D2). However, the most obvious positive staining for OCN was found in the PHMBD group. Analysis of bone regeneration in calvarial defects indicated that co-delivery of DMOG and rhBMP-2 can synergistically increase bone regeneration. A similar tendency was exhibited for the angiogenic marker CD31 (Fig. 9E–H). Positive brown staining for CD31 was more obvious in the PHMD (Fig. 9G1,G2) and PHMBD groups (Fig. 9H1,H2) compared with the PHMB (Fig. 9F1,F2) and PHMG groups (Fig. 9E1,E2) and, interestingly, the most obvious positive staining occurred in the PHMBD group in spite of the lower effectiveness of the PHMB group. The analysis of calvarial defects indicated that DMOG and rhBMP-2 released in the PHMBD group can synergistically improve neovascularization.

## Discussion

In this study, we have demonstrated that the synergistic effects of DMOG and rhBMP-2 released from PHMG composite scaffolds can repair critical-sized bone defects by enhancing bone regeneration and neovascularization in rats. In the field of tissue engineering, it is essential to create novel scaffolds to repair bone defects. Accumulated evidence shows that angiogenesis plays a critical role in skeletal development and repair^5^,^23^, and BMP-2 is well known as a strong inducer of bone formation. Previously, Zhao *et al*.^24^ suggested that the presence of a sulfated chitosan coating could enhance the release profile of a calcium-deficient hydroxyapatite/BMP-2 composite and, in turn, promote new bone formation in a rat cranial defect model. Small-molecule drugs and some recombinant proteins are appealing for regenerative medicine applications because many exhibit extended *in vivo* stability and a limited period of action. They are inexpensive, and scalable production is possible^10^,^11^,^25^. Angiogenesis is an essential stage of bone healing as restoration of blood flow provides nutrients and renewable autologous cells to heal the defects^26^. A number of small-molecule drugs have been investigated for bone regeneration and angiogenesis. For example, recent research indicated that DMOG can induce a pro-angiogenic effect by stabilizing HIF-1a and initiating the subsequent activation of VEGF^27^,^28^. It is known that the mesoporous properties of biomaterials are of great importance for influencing the loading and release of drugs^8^,^9^. In this study, we applied PHMG composite scaffolds as a matrix, as well as releasing rhBMP-2 and DMOG simultaneously to stimulate bone regeneration and angiogenesis in bone defects.

It is rational that rhBMP-2 released in PHMG composite scaffolds significantly promoted osteogenic differentiation by enhancing the bone-related gene expression (ALP, COL-1, and RUNX-2) of hBMSCs (Fig. 4A–C), and DMOG released in PHMG composite scaffolds clearly increased the expression levels of HIF-1a and VEGF (Fig. 4D,E). Delivery of rhBMP-2 and DMOG in PHMG composite scaffolds clearly upregulated the expression levels of the bone-related genes (ALP, COL-1, and RUNX-2) as well as HIF-1a and VEGF. These results suggest that concurrently delivery of rhBMP-2 and DMOG in PHMG composite scaffolds might have synergistic effect on osteogenic and angiogenic differentiation of hBMSCs. The related molecular mechanism is not fully understood. It has been reported in some studies^29^,^30^ that RUNX-2 may serve as a transcription factor to induce VEGF transcription. Other researchers^31^,^32^,^33^ have suggested that VEGF may stimulate osteogenic differentiation and that there is therefore a positive feedback loop between the VEGF and RUNX-2 proteins. In this study, DMOG stimulated VEGF expression and rhBMP-2 upregulated RUNX-2 expression. Hence we thought that the combination of rhBMP-2 and DMOG could synergistically promote osteogenic differentiation and angiogenic expression through the positive feedback loop between VEGF and RUNX-2.

Our *in vivo* results showed that rhBMP-2 released in PHMG composite scaffolds enhanced new bone formation to a greater extent in the PHMG and PHMD groups, but the highest values of BMD and BV/TV ratio measured by micro-CT quantitative analysis were observed in the PHMBD group. The histomorphometric analysis based on Van Gieson’s picrofuchsin staining also showed that the largest area of newly-formed bone was found in the PHMBD group, and this was confirmed by the fluorescent labeling results. OCN acts as the key osteogenesis-related marker in the process of bone regeneration^34^,^35^,^36^. The IHC staining results validated that OCN was highly expressed in the PHMBD group compared with the other groups. Microfil^®^ perfusion and CD31 IHC were used to evaluate the neovascularization of defects. The results demonstrated that DMOG released in PHMG composite scaffolds enhanced neovascularization to a greater extent in the PHMG and PHMB groups, which was consistent with the research of Min *et al*.^22^. However, the co-delivery PHMBD group was demonstrated significantly more neovascularization than that in the PHMD group. The above results indicate that rhBMP-2 can promote new bone formation and DMOG can stimulate the generation of more new microvessels, thus providing a larger blood supply to enhance the ability to repair bone defects. As a consequence, rhBMP-2 and DMOG released in PHMG composite scaffolds can synergistically improve repair of bone defects.

## Conclusions

In this study, PHMG composite scaffolds were fabricated to deliver DMOG and rhBMP-2 simultaneously. Respective roles of rhBMP-2 and DMOG in the co-delivery system were verified. Notably, the interest arises from the reciprocal effects between BMP-2 and DMOG. In spite of the disparate pathway, rhBMP-2 can stimulate angiogenic capacity of DMOG; while DMOG can promote osteogenic capacity of rhBMP-2 under the concurrent conditions. Attribute to the synergistic effects, induction of both osteogenic and angiogenic differentiation of hBMSCs were amplified. As a result, this strategy can significantly improve the repair of critical-sized bone defects in rats via the synergistic effect of simultaneously stimulating osteogenic activity and timely provision of a blood supply. Based on the above results, we therefore envisage that the combinational application of rhBMP-2 and DMOG will be promising for bone regeneration. The relevant mechanism and signaling pathway of reciprocity is under study.

## Materials and Methods

All methods were carried out in accordance with relevant guidelines and regulations of the Research Ethics Committee of the Shanghai Sixth People’s Hospital-affiliated Shanghai Jiao Tong University, all experimental protocols were approved by the Research Ethics Committee of the Shanghai Sixth People’s Hospital-affiliated Shanghai Jiao Tong University.

### Materials

PHBHHx (molecular weight 200,000 Da) was purchased from Shantou Lianyi Biotech Company (Shantou, China). P123 (EO_20_PO_70_EO_20_) was obtained from Sigma-Aldrich (St Louis, MO, USA). Tetraethyl orthosilicate (TEOS), triethyl phosphate (TEP), and Ca(NO_3_)_2_•4H_2_O were obtained from Shanghai Ling Feng Chemical Reagent Co. Ltd. (Shanghai, China). RhBMP-2 was provided by Shanghai Rebone Co., Ltd. (Shanghai, China). DMOG was obtained from Santa Cruz Biotechnology, Inc. (Santa Cruz, CA, USA). All reagents were analytical grade and were used as received.

### Preparation and characterization of PHMG composite scaffolds

MBG was synthesized as previously described^37^,^38^. Briefly, P123 (4.0 g), TEOS (6.7 g), Ca(NO_3_)_2_•4H_2_O (1.4 g), TEP (0.36 g; molar ratio of Si:Ca:P 80:15:5), and 0.5 M HCl (1.0 g) were dissolved in ethanol (60 g) and stirred at room temperature for 1 day. The resulting sol was put in a Petri dish to undergo an evaporation-induced self-assembly (EISA) process. The dried gel was calcined at 700 °C for 5 h to obtain the final MBG products.

Porous PHMG scaffolds were prepared by freeze-drying and a particulate leaching technique. Briefly, PHBHHx was dissolved in 1,4-dioxane (10% w/v) and MBG powder was added to the PHBHHx solution. The ratio of PHBHHx: MBG was 4:1 (w/w). Subsequently, sieved salt particles 400–800 μm in diameter were dispersed in the mixture. The ratio of PHBHHx: salt was 1:8 (w/w). The blend was mixed briefly to form homogeneous slurry and cast into Teflon™ molds with an inner diameter of 5 mm and a height of 2 mm. After being frozen at −20 °C for 12 h, the scaffolds were lyophilized for 12 h and the salt was then completely leached out in deionized water. The scaffolds were freeze-dried again, and then sterilized using gamma irradiation.

The mesoporous structure of MBG was confirmed using transmission electron microscopy (TEM; JEM-2100, JEOL, Tokyo, Japan). Brunauer–Emmet–Teller (BET) and Barrett–Joyner–Halenda analyses were performed to determine the surface area and pore parameters using a Micromeritics porosimeter (Tristar 3000; GA, USA). The hierarchical microstructure of the 3D scaffold was characterized by scanning electron microscopy (SEM; S-3400, Hitachi, Tokyo, Japan) and micro-CT (mCT-80; Scanco Medical AG, Bassersdorf, Switzerland).

The PHMG scaffold with a 5 mm in diameter and 5 mm height were measured under compression using a universal testing machine (HY-0230, Shanghai Hengyi Testing Instruments Co.,Ltd, China) at a cross-head speed of 1 mm/min. At least five replicates were carried out for each group, and the results were expressed as means ± standard deviation (means ± SD).

### Loading and release of DMOG and rhBMP-2

As previously described^17^,^22^, DMOG powder was first dissolved in phosphate-buffered saline (PBS) at a concentration of 1 mg/mL. DMOG solution (50 μL) was added dropwise onto each PHMG composite scaffold, and the scaffolds were then freeze-dried. For the DMOG-release analysis, the DMOG-loaded scaffolds were incubated in 2 mL PBS at 37 °C on an orbital shaker at 100 rev/min. At scheduled time-points, 1 mL of PBS was collected, and the same volume of fresh medium was then added to the vials. Release of DMOG was monitored by ultraviolet spectrometry (SpectraMax M2; Molecular Devices Inc., Sunnyvale, CA USA) at a wavelength of 230 nm. The cumulative release of DMOG from the PHMG scaffolds was calculated. Parallel experiments were carried out in triplicate and the average value was calculated for each specimen.

For rhBMP-2 loading, 25 μL of rhBMP-2 solution (2 mg/mL) was used^38^. Similar procedures were conducted as described above. Released BMP-2 was collected, replenished in 100 μL at the specified intervals and determined using an enzyme-linked immunosorbent assay kit (Beyotime, China). The cumulative release rate of rhBMP-2 from the PHMG scaffolds was calculated. Three replicates were tested for each sample.

### Cell cultures

The murine-derived macrophage cell line RAW 264.7 cells and human bone marrow stromal cells (hBMSCs) were used in this study. All experiments of cells were performed in accordance with relevant guidelines and regulations of the Research Ethics Committee of the Shanghai Sixth People’s Hospital-affiliated Shanghai Jiao Tong University. RAW 264.7 cell cultures were maintained in Dulbecco’s Modified Eagle Medium (DMEM, Life Technologies, Carlsbad, California, USA) containing 10% foetal bovine serum(FBS, Thermo Scientific, Massachusetts, USA), and 100 U/mL penicillin and 100 mg/L streptomycin (Hyclone) at 37 °C in a humidified CO_2_ incubator. hBMSCs were obtained from three donors who gave their written informed consent. Briefly, marrow was extracted from the femoral midshaft and then suspended in minimum essential medium containing 10% fetal bovine serum (Hyclone; GE Healthcare, Little Chalfont, UK), 100 U/mL penicillin and 100 mg/L streptomycin. Subsequently, the non-adherent cells were discarded; the adherent cells converged to 80–90% confluence and were then replated as passage one (P1) cells. P3 cells were used for experiments. A density of 1 × 10^5^ cells/mL was used in the cellular tests.

### Cell seeding on porous PHMG composite scaffolds

Prior to seeding cells, the prefabricated PHMG scaffolds were sterilized using gamma irradiation. Cell suspension (100 μL) was added to four groups at a density of 1 × 10^4^ cells/scaffold. After 4 h, 100 μL of culture medium was carefully added to the base of the culture plate until the scaffold was covered with sufficient culture medium.

### Cell attachment and viability

The viability of visualized cells was evaluated using a live/dead assay kit (Abcam, Cambridge, UK) following the standard protocol. Briefly, the RAW 264.7 cells and scaffold constructs were first washed twice with PBS and then incubated in standard working solution at room temperature for 10 min. The constructs were then washed twice with PBS and observed under a confocal laser scanning microscope (CLSM; NikonA1R; Nikon, Japan).

To investigate the morphology of RAW 264.7 cells on scaffolds, the cell/scaffolds were collected after being cultured for 7 days, rinsed in PBS and then fixed with 2.5% glutaraldehyde in PBS for 1 h. Then cell/scaffolds were washed with buffer containing 4% (w/v) sucrose in PBS and post-fixed in 1% osmium tetroxide in PBS, and then were dehydrated in a graded ethanol series (30, 50, 70, 90 and 100%) for 10 min each and twice in absolute ethanol, freeze dried, coated with gold, and examined by SEM.

### Real-time quantitative reverse transcription polymerase chain reaction (qRT-PCR) analysis

Osteogenic and angiogenic gene expressions, including those of runt-related transcription factor 2 (RUNX-2), collagen type I (COL1), alkaline phosphatase (ALP), HIF-1a, and VEGF, were assessed by RT-qPCR. Total ribonucleic acid (RNA) from cells of the four groups was extracted and harvested using the Trizol method (Invitrogen, Carlsbad, CA, USA) for 7 days. The RNAs were reverse-transcribed into complementary deoxyribonucleic acid (cDNA) and amplified using a One Step SYBR RT-PCR kit (Takara Bio Inc., Shiga, Japan). The amplification of the cDNA was performed using SYBR Premix Ex Taq (Takara) in combination with a ViiA7 Real-Time PCR System (Life Technology, ABI). β-actin was used as the internal control for PCR amplification. Each sample was analyzed in triplicate. Primer information is given in Table 1.

### Western blotting analysis

Western blotting analysis was performed to detect ALP, RUNX-2, COL1, HIF-1α, and VEGF at the protein level. Briefly, 7 days after seeding onto the scaffold, whole-cell lysates were obtained by lysing the cells in a radio immunoprecipitation assay buffer (Bio-Rad, Hercules, CA, USA). Protein from each group was separated by 9% sodium dodecyl sulfate–polyacrylamide gel electrophoresis, and then transferred to a 0.22-mm polyvinylidine difluoride membrane (Millipore; Billerica, MA, USA). After being blocked with 5% non-fat dried milk, the membranes were incubated with the appropriate primary antibody at a dilution of 1:1,000, followed by the respective secondary antibody at a dilution of 1:5,000. The bands were imaged. This experiment was repeated three times.

### Animal experiments

Animal experiments were approved by the Research Ethics Committee of the Shanghai Sixth People’s Hospital-affiliated Shanghai Jiao Tong University, and performed in accordance with the Care and Use of Laboratory Animals protocols.

Briefly, 24 mature Sprague–Dawley (SD) male rats (mean body weight 250–300 g) were provided with sterilized food and water and housed in a barrier facility with a 12-h light/dark cycle. These rats were randomly divided into four groups, each containing six rats: PHMG, PHMB, PHMD, and PHMBD. For the surgical procedure, as previously described^39^, the animals were anesthetized by intraperitoneal injection of chloral hydrate (4%; 9 mL/kg body weight) and all operations were performed under sterile conditions. A 1.5-cm sagittal incision was made in the scalp and the calvarium was exposed by blunt dissection. Two critical-sized calvarial defects with a bilateral diameter of 5 mm were created using a dental trephine, and the scaffolds were then implanted into the defects. Following the operation, the animals received intramuscular antibiotic injections, were allowed free access to food and water and were monitored daily for potential complications. Eight weeks after the operation, the rats were killed by an overdose of anesthetics and their craniums were harvested and fixed in a 4% paraformaldehyde solution buffered with 0.1 M phosphate solution (pH 7.2) overnight before further analysis.

### Iconographic analysis

All the harvested specimens were examined using the mCT-80 system to evaluate new bone formation within the defect region. Briefly, the undecalcified samples were scanned at a resolution of 18 μm and decalcified samples perfused with Microfil^®^ were scanned at a resolution of 9 μm. After 3D reconstruction, the bone mineral density (BMD) and bone volume fraction (bone volume/total volume [BV/TV]) in the defect regions were used to calculate new bone formation using the auxiliary software of the mCT-80 system^40^.

### Sequential fluorescent labeling

2, 4, and 6 weeks after the craniotomies, the SD rats were intraperitoneally injected with tetracycline (TE; 25 mg/kg body weight), alizarin red (AL; 30 mg/kg body weight), and calcein (CA; 20 mg/kg body weight), respectively. Mineralized tissues were observed by means of trichromatic sequential fluorescent labeling^41^.

### Microfil^®^ perfusion

To compare blood vessel formation *in vivo*, animals were perfused with Microfil^®^ (Flowtech, Carver, MA, USA) after euthanasia 8 weeks post-operatively. In brief, the rib cage was opened, and the descending aorta clamped. An angiocatheter was used to penetrate the left ventricle. After the inferior vena cava was incised, 20 mL of heparinized saline was perfused, followed by perfusion of 20 mL of Microfil^®^ at a flow rate of 2 mL/min. Finally, the rats were maintained at a temperature of 4 °C for 1 day^42^.

### Histological analysis

One part of each cranium was dehydrated in a graded alcohol series ranging from 70% to 100%, and then embedded in polymethylmethacrylate. After hardening, longitudinal sections were cut into 150–200 μm slices using a microtome (Leica Microsystems Ltd, Wetzlar, Germany), glued onto a plastic support and then polished to a final thickness of approximately 50 μm. First, the sections were examined using a CLSM (Leica, Heidelberg, Germany) for fluorescent labeling. Then, new bone formation and mineralization were quantified at four locations that equally divided the defect site between the two ends of the longitudinal sections. The mean value of the four measurements was calculated to give average values for each group. The sections were then stained with van Gieson’s picrofuchsin to evaluate new bone formation^6^. The area of new bone formation was quantitatively evaluated at four random sections using Image Pro 5.0 software (Media Cybernetics, Rockville, MD, USA).

### Immunohistochemical (IHC) analysis

The other part of each cranium was decalcified for approximately 2 weeks, dehydrated using a graded alcohol series, embedded in paraffin and sectioned into 5-μm sections. Osteocalcin (OCN) IHC was performed to evaluate osteogenesis, and CD31 IHC was used to detect angiogenesis in specimens^42^.

### Statistical analysis

All of the above data were analyzed as mean ± standard deviation. Differences between groups were calculated using one-way analysis of variance. The statistical analysis was conducted using SPSS 17.0 software (SPSS Inc., Chicago, IL, USA). *P* < 0.05 was considered to indicate statistical significance.

## Additional Information

**How to cite this article:** Qi, X. *et al*. Synergistic effects of dimethyloxallyl glycine and recombinant human bone morphogenetic protein-2 on repair of critical-sized bone defects in rats. *Sci. Rep.* **7**, 42820; doi: 10.1038/srep42820 (2017).

**Publisher's note:** Springer Nature remains neutral with regard to jurisdictional claims in published maps and institutional affiliations.

## Figures and Tables

**Table 1:** Sequences of primers used in RT-qPCR.

Gene	Primer sequences(F, forward; R, reverse; 5′−3′)
ALP	F: GTTTTCTGTTCTGTAAGACGGG
	R: GCCGTTAATTGACGTTCCGA
RUNX-2	F: CCGAGCTACGAAATGCCTCT
	R: GGACCGTCCACTGTCACTTT
COL1-A1	F: ACATGTTCAGCTTTGTGGACC
	R: AGGTTTCCACGTCTCACCAT
HIF-1a	F: CAAAGCTCATCCAAGGAGCC
VEGF	R: GCTGCAGTAACGTTCCACTT
	F: ACTGGACCCTGGCTTTACTG
	R: CTGGAAGATGTCCACCAGGG
